# Gene-Disease Network Analysis Reveals Functional Modules in Mendelian, Complex and Environmental Diseases

**DOI:** 10.1371/journal.pone.0020284

**Published:** 2011-06-14

**Authors:** Anna Bauer-Mehren, Markus Bundschus, Michael Rautschka, Miguel A. Mayer, Ferran Sanz, Laura I. Furlong

**Affiliations:** 1 Research Programme on Biomedical Informatics (GRIB), IMIM (Hospital del Mar Research Institute), Universitat Pompeu Fabra, Barcelona, Spain; 2 Institute for Computer Science, Ludwig-Maximilians-University Munich, Munich, Germany; Memorial Sloan Kettering Cancer Center, United States of America

## Abstract

**Background:**

Scientists have been trying to understand the molecular mechanisms of diseases to design preventive and therapeutic strategies for a long time. For some diseases, it has become evident that it is not enough to obtain a catalogue of the disease-related genes but to uncover how disruptions of molecular networks in the cell give rise to disease phenotypes. Moreover, with the unprecedented wealth of information available, even obtaining such catalogue is extremely difficult.

**Principal Findings:**

We developed a comprehensive gene-disease association database by integrating associations from several sources that cover different biomedical aspects of diseases. In particular, we focus on the current knowledge of human genetic diseases including mendelian, complex and environmental diseases. To assess the concept of modularity of human diseases, we performed a systematic study of the emergent properties of human gene-disease networks by means of network topology and functional annotation analysis. The results indicate a highly shared genetic origin of human diseases and show that for most diseases, including mendelian, complex and environmental diseases, functional modules exist. Moreover, a core set of biological pathways is found to be associated with most human diseases. We obtained similar results when studying clusters of diseases, suggesting that related diseases might arise due to dysfunction of common biological processes in the cell.

**Conclusions:**

For the first time, we include mendelian, complex and environmental diseases in an integrated gene-disease association database and show that the concept of modularity applies for all of them. We furthermore provide a functional analysis of disease-related modules providing important new biological insights, which might not be discovered when considering each of the gene-disease association repositories independently. Hence, we present a suitable framework for the study of how genetic and environmental factors, such as drugs, contribute to diseases.

**Availability:**

The gene-disease networks used in this study and part of the analysis are available at http://ibi.imim.es/DisGeNET/DisGeNETweb.html#Download.

## Introduction

For many years, scientists have been trying to understand the molecular and physiopathological mechanisms of diseases in order to design new preventive and therapeutic strategies. The combination of experimental and computational methods has led to the discovery of disease-related genes [1], [2]. A well-known example is Phenylketonuria, where the function of the gene encoding the PAH enzyme was studied with respect to the mechanism of the disease [3]. However, we are still far from fully understanding disease causation, especially regarding complex diseases such as cancer [2]. Even for mendelian diseases this is not fully achieved because phenotypic outcome cannot be predicted solely based on the genotype [3]. It has become evident, that many human diseases cannot be attributed to malfunction of single genes but arise due to complex interactions among multiple genetic variants [4]. Moreover, influences of environmental factors, infectious agents or drugs have to be considered when studying the occurrence and evolution of a disease. In complex diseases, alterations in several genes can make subtle contributions to the susceptibility of a particular individual. At the end of the day, how a disease is caused and thus how it can be treated can only be studied on the basis of the entire body of knowledge including all genes that are associated with the disease and their interactions through biological pathways. However, with the unprecedented wealth of information available, it is extremely difficult to obtain a complete picture of the genetic basis of diseases. In order to obtain such a complete picture, data integration from different sources is required. This is of special interest considering the fact that individual researchers are often restricted to so called knowledge pockets [5] covering only a small fraction of all available knowledge that is spread all over the literature or various databases. This fragmentation of information clearly hampers our understanding of the molecular processes underlying human diseases.

In the 60s, Dr. McKusick, the initiator of the Online Mendelian Inheritance in Man (OMIM) database, started collecting information about genes and their association to diseases first as a book and later as a database. OMIM has become a highly popular source in medical genetics [6]. It traditionally focused on mendelian diseases and later started to include complex diseases as well. In the last years, other databases have been built, among them the Pharmacogenomics Knowledge Base (PHARMGKB) specialized on the knowledge about genes that are involved in modulating drug response [7] or the Comparative Toxicogenomics Database (CTD) focused on the effect of environmental chemicals on human disease [8]. Each of the databases focuses on different aspects of phenotype-genotype relationships. However, due to the fast increase of literature in the life science domain, no one (not even expert curators of such databases) can keep track of the relevant knowledge that is regularly published [5]. Here, text-mining has evolved as a useful tool to automatically extract the information about the relationships between biomedical entities reported in the literature (for a recent overview see [9]). In this work we developed a comprehensive database of human gene-disease associations by integrating both, information from different databases and from literature, in order to bridge the gaps between the aforementioned knowledge pockets. The resulting database (DisGeNET database) comprises the whole spectrum of human diseases with genetic origin, including mendelian, complex and environmental diseases, and represents, to the best of our knowledge, the most complete view on human gene-disease associations that is currently publicly available.

Many phenotypically similar diseases are caused by functionally related genes, such as Stickler, Marshall and OSMED syndromes [10], [11], [12], or several forms of human ataxias [13]. Hence, many diseases are caused by dysfunction of so-called functional modules [13], [14], [15], [16]. Functional modules can be defined as a group of cellular components and their interactions that carry out a specific biological function [17]. These functional modules can be either physically constrained like the ribosome or spread over the cell like a signal transduction pathway. Alterations in the individual components of a specific functional module can result in similar disease phenotypes. Accordingly, several studies proposed the concept of modularity for human genetic diseases taking malformation syndromes as examples [18], [19], [20].

The concept of modularity of human diseases is the basic assumption of several methods for the identification and prioritization of candidate disease genes [2]. Surprisingly, the concept of modularity of human diseases has not been evaluated systematically for all the diseases, except for the ones available at OMIM (see [21] for a recent review) and individually for some diseases (see below). Moreover, while the concept of modularity proved to be useful when studying mendelian or oligogenic traits, there is still limited evidence for its applicability for complex traits [20]. Most of the current approaches to identify disease relevant modules focus on individual diseases [13], [14], [15], [16]. A different strategy was proposed by [22], who performed a global analysis of a human gene-disease associations based on the OMIM database to show that gene products related to the same disease have a higher likelihood to physically interact [22].

In this article, we pick up the concept of modularity of human genetic diseases with the aim of assessing it for the whole spectrum of diseases with genetic origin, which had not been studied before. For this purpose we use networks, which allow the representation of the relationships between biomedical entities [22], [23], [24] and the subsequent analysis of emergent properties [25]. It has been observed that topological properties of biological networks differ from those in random networks [26]. Moreover, it has been shown how cluster analysis of protein-protein interaction networks can be used to identify functional modules [23], [27]. Hence, we use topological and functional analysis of gene-disease association networks to assess the modularity of mendelian, complex and environmental diseases. Our results indicate that for most human diseases functional modules do exist. This is also observed for groups of diseases sharing gene associations. Moreover, our results point out that most human diseases are associated with more than one biological process. This contrasts with previous observations based on OMIM data. We show in several case studies how the network representation of human genetic diseases and the adjacent detection of functional modules can be used not only to shed light on the molecular basis of human diseases but also to gain a better understanding of the influence of environmental factors, including drugs, on human health. Moreover, our analysis confirms the need of integrating human gene-disease associations from various sources. To enforce research in this field, we make all gene-disease networks publicly available as SQLite database for computational access, as well as through DisGeNET, a plugin for Cytoscape [28] to access and analyze our data.

## Results and Discussion

### Topological network analysis

**Figure 1:**
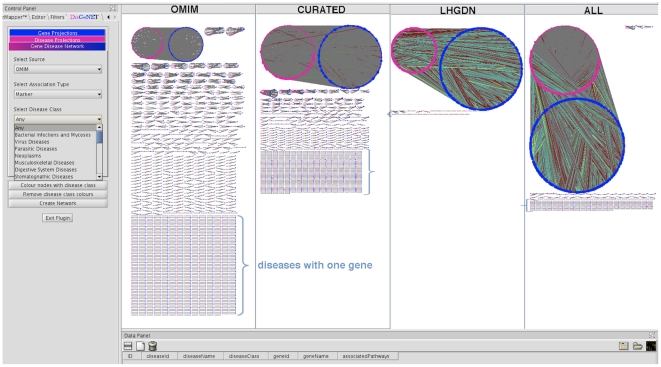
Cytoscape screenshot depicting the four gene-disease networks. Gene (blue) and disease (magenta) nodes are connected by edges in different colors corresponding to the type of association in our gene-disease association ontology. Grey represents Marker association, red denotes GeneticVariation, blue corresponds to Therapeutic class, green to RegulatoryModification.

Previous work has shown that topological network analysis of gene-disease associations uncovers important properties of the nature of mendelian diseases [22]. We used four different bipartite networks called OMIM, CURATED, LHGDN and ALL (see Methods) to study human diseases at a global scale, including mendelian, complex and environmental diseases. By comparing the four networks we show that our integration effort results in a huge increase in coverage of (i) diseases, (ii) genes and (iii) their associations compared to the individual data sources (see Fig. S2). The overlap among databases is surprisingly small (see Fig. S3) confirming the existence of the aforementioned knowledge pockets and highlighting the need of integrating different data sources. We demonstrate how the integration can close knowledge gaps in Case study 2 (section Case studies). Here, the association between MITF, a transcription factor regulating the expression of the TYR gene, and Melanoma [29], [30] was not found in any of the curated databases but was present in the text-mining derived network. The more data sources are considered the denser the networks become, indicating that many more diseases share genetic origin than reflected in a single source (Fig. 1). In OMIM, most diseases are associated with few genes. Contrasting, in the other networks most diseases are part of a large connected component that increases noticeably when integrating more data. Concomitantly the number of diseases associated with only one gene decreases (see curly brackets in Fig. 1) suggesting that most diseases are associated with more than one gene, even for mendelian diseases. Note that this is already observed in the network CURATED. Previous studies have reported that for some mendelian disorders, such as Phenylketonuria, the observed phenotype is the result of the combined effect of a primary gene and other genes that act as modifiers [3], [31]. Our results indicate that the same phenomenon might be in place for other mendelian disorders. In addition, our results support the hypothesis that the distinction between mendelian and multiple gene disorders is rather artificial and that the influence of several genes, including the so-called modifier genes, should be studied in more detail for mendelian disorders [3], [31].

We furthermore show that the degree distributions of diseases and genes are different from the degree distribution of random networks, but none of them follows a power law distribution (see Fig. S5). Although many early studies about the topology of biological networks proposed power-law behavior, recent re-evaluations indicate that this is not always the case and other models need to be considered [32], [33]. Nevertheless, there are two main trends visible. Both, the number of hubs and the average degree size increase dramatically due to the integration process. For the gene nodes, the average degree ranges from 1.6 in OMIM to 5.6 in ALL and for the disease nodes, from 1.5 in OMIM to 10.1 in ALL (see Fig. S5). The degree of a disease node represents the number of associated genes and hence can be used as a measure for the locus heterogeneity of the disease. Overall, there is a dramatic increase in the maximum locus heterogeneity. With respect to the genes, the increase in the node degree is less dramatic but still visible (see Fig. S5).

To study the diseases and disease-related genes in more detail, we also generated gene and disease centric views of the data by projecting the bipartite gene-disease networks to monopartite networks. Similarly to the observations in the bipartite networks, the node degrees increase dramatically when integrating more data. All in all, our results suggest a much higher level of interrelation of human diseases than observed by solely considering a single data source (e.g. OMIM).

In summary, the network analysis of our integrated database points out that data integration is needed to obtain a comprehensive view of the genetic landscape of human diseases and that the genetic origin of mendelian, complex and environmental diseases is much more common than expected.

### Functional network analysis

#### A. Functional analysis at the level of individual diseases

Several studies based on the OMIM database indicated that for diseases with high locus heterogeneity the associated genes are involved in the same biological process, supporting the concept of functional modules associated with disease [22], [34], [35]. This concept of modularity is often assumed to be valid for all human diseases; however it has not been tested before for monogenic, complex and environmental diseases in a systematic manner. The DisGeNET database represents an appropriate resource to perform such an evaluation. Thus, we wanted to assess if the disease-related genes in our integrated data set were involved in the same biological processes. In other words, we wanted to study if the concept of modularity applies to the whole spectrum of human genetic diseases. For this purpose, we calculated pathway homogeneity with the following average values: 0.77 (sd 0.26) in OMIM, 0.67 (sd 0.27) in CURATED, 0.56 (sd 0.24) in LHGDN and 0.59 (sd 0.25) in ALL. Interestingly, in comparison to the OMIM dataset, the homogeneity values decreased for the larger networks (CURATED, LHGDN, ALL).

**Figure 2:**
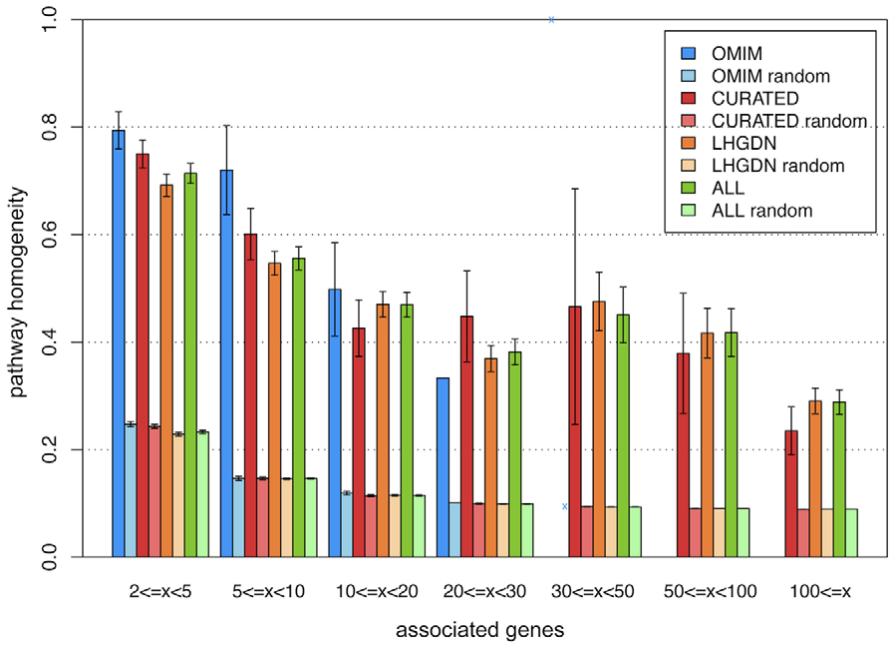
Pathway homogeneity for individual diseases. Mean pathway homogeneity values of single diseases and random controls are plotted for all four networks binned by the number of associated gene products per disease. Pathway homogeneity values range from 0 to 1, where 1 means that all gene products associated with the disease are annotated to the same pathway. Confidence intervals of 95% were added to allow comparison of real to random values. For OMIM, there are only two diseases with more than 30 gene products annotated, both with a pathway homogeneity of 1.

To assess if the decrease of homogeneity values results from the integration effort, we studied the dependency of homogeneity values on the number of associated genes. Interestingly, for all data sources, even for OMIM, the homogeneity decreases with increasing number of associated gene products (r = −0.25) (see Fig. 2). Nevertheless, homogeneity values are significantly higher than for random controls (see Fig. 2), indicating that genes related to the same disease are more likely to be involved in the same biological pathways than randomly selected disease genes. For instance, in CURATED, diseases with two to five annotated gene products have an average pathway homogeneity of 0.75 (sd 0.26), suggesting that on average 75% of the gene products are annotated to the same pathway, while this value decreases to 0.38 (sd 0.15) if 50 to 100 gene products are annotated to the disease (see Fig. 2). For diseases with two to five annotated gene products, approximately 70% of them participate in the same pathway for all data sources. On the other hand, for diseases with more than 10 gene products annotated, it is more likely that more than one pathway is involved. Moreover, it is striking that although the text-mining derived network is very dense with an average of 18.7 genes per disease, the homogeneity values still differ significantly from random, with values comparable to the ones in CURATED. The observed decrease of homogeneity was observed for all databases including OMIM suggesting that it is not related to the integration effort but arises due to increased locus heterogeneity of diseases. This observation should be taken into account in future studies of genetic diseases.

Strikingly for all datasets the homogeneity values differ significantly from random controls supporting that for most diseases functional modules exist. Thus, the concept of modularity is observed for mendelian diseases, in agreement with previous reports [13], [18], [22], [34], [35], but also for complex and environmental diseases. Moreover, the analysis shows that a core set of biological pathways is associated with most human diseases. Similar observations were reported for different cancer types, such as for Pancreatic Cancer [14] or Glioblastoma [36]. Our results show that this is also the case for the diseases present in DisGeNET. Similar results were recently reported by [37] showing on average 12 pathways associated with a disease, using a database based on co-occurrence of genes and diseases in the literature.

### B. Functional analysis at the cluster level

#### B.1. Functional analysis of disease clusters

We applied the MCL graph-clustering algorithm (see Methods) to identify highly connected units, so called clusters, in the disease projection networks and then tested if the disease clusters are associated with functionally relevant modules. For this purpose, we determined if the genes associated with the disease clusters are more likely to participate in the same biological processes than randomly selected genes by calculating pathway homogeneity (see Fig. S6). On average, pathway homogeneity is 0.68 (sd 0.24) for OMIM and 0.59 (sd 0.25) for CURATED, suggesting that in these datasets 60–70% of the gene products belonging to a disease cluster participate in the same pathway. For the more populated networks (LHGDN and ALL) the average pathway homogeneity values of disease clusters decreases to approximately 0.48 (sd 0.23). However, we again observe a decrease of the pathway homogeneity with increasing number of gene products annotated to the diseases clusters (r = −0.26) (see Fig. S6). A more detailed analysis of the disease clusters revealed that the majority has medium pathway homogeneity values and there are only few extremely homogeneous or heterogeneous disease clusters. Overall, the results for single diseases and disease clusters are similar. Hence, for most diseases and even for clusters of diseases, more than one biological pathway is associated. It can be argued that for these diseases cross-talks of pathways could play an important role. For instance, the cross-talk between Integrin and TGF-b pathways has been found to be related to several human pathologies including systemic sclerosis, idiopathic pulmonary fibrosis, chronic obstructive pulmonary disease and cancer [38].

#### B.2. Functional analysis of gene clusters

Studies focused on a specific disease presented evidence arguing for a modular nature of the disease, especially for congenital malformations and related syndromes [13], [18]. In the previous sections, we evaluated from a disease centric view if functional modules exist for human genetic diseases. In this section, we assess the modularity from the gene centric view by evaluating if groups of phenotypically related genes represent functional modules in the cell. For this purpose we analyzed the functional annotations of gene products in clusters derived from applying the graph-clustering algorithm on the gene projection networks (see Methods).

Gene products can be functionally related to each other in different ways: by means of direct, physical protein-protein interactions or by more indirect associations as observed between enzymes in the context of a metabolic pathway.

**Figure 3:**
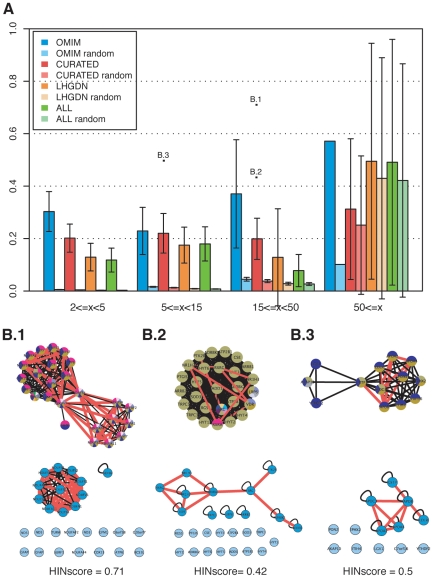
HINscores for phenotypically derived gene clusters. A: Mean HINscores plotted for different cluster sizes for all networks and random controls. B: Selected gene clusters denoted as B.1, B.2, B.3 and their corresponding HIN subgraphs from the CURATED dataset. In the phenotypically derived gene clusters (upper part) red edges represent physical interactions among the gene products. In the HIN subgraphs (lower part), red edges denote phenotypic relationship among the corresponding genes. Nodes in light blue belong to the phenotypically derived gene clusters that are not present in HIN. B.1 is associated with mitochondrial respiratory chain deficiencies, Leigh and Alexander Disease. B.2 corresponds to Hypertension and Cardiovascular Diseases. B.3 represents different types of Hyperlipoproteinemia. Nodes are colored according to their disease class (see Fig. S4).

First we assessed to which degree the proteins encoded by the genes in the clusters directly interact in the cell. For this purpose, we used a recently published human interaction network (HIN) based on protein-protein and signaling interactions [36] and calculated HINscores for the gene clusters (see Methods). The HINscore ranges from 0, where none of the gene products in a cluster directly interacts in HIN, to 1, if all gene products physically interact in HIN and hence the gene cluster represents a biologically functional module. Therefore, the HINscore can be used to measure the modularity of human diseases in our networks. For CURATED and OMIM, clusters including less than 50 nodes show HINscores significantly higher than for random clusters, while for the other networks the difference is significant for clusters of less than 15 nodes (see Fig. 3A). Fig. 3B illustrates some selected clusters from CURATED with high HINscores. For instance, cluster B.1 contains genes mainly associated with mitochondrial complex I deficiency (genes in the lower right part of the gene cluster), and Leigh and Alexander diseases (genes in the upper left part of the gene cluster). The latter are neurometabolic disorders that result from defects in the mitochondrial respiratory chain. Genes associated with these diseases encode proteins that form a physically interacting module as illustrated by the HIN subgraph. Other examples of clusters with high HINscore are related to peroxisomal disorders (e.g. Zellweger syndrome), different types of anemia (Diamond-Blackfan anemia or Heinz body anemia) or Walker-Warburg and Fukuyama syndromes. Thus, the HINscore can be used to identify phenotypically derived gene clusters, in which direct interactions between the gene products might play an important role.

Second, we evaluated the indirect relationships between disease gene products. For this purpose we calculated pathway homogeneity of the phenotypically related gene clusters. In CURATED, 60% of the clusters have pathway homogeneity values <0.75. Hence for more than half of the clusters, there are at least two pathways annotated. In CURATED, the average pathway homogeneity for clusters smaller than 50 nodes is significantly higher than for randomly selected clusters (see Fig. S7). For clusters larger than 50 nodes the results are not significantly different from random controls; however, such clusters are underrepresented in our dataset. Moreover, similar to individual diseases and disease clusters, we observe that the homogeneity decreases with increasing size of the cluster for all data sets (r = −0.20).

### C. Summary and outlook

Most of the gene clusters (72%) in CURATED are of size smaller than 15. Interestingly, for such clusters, HINscores and homogeneity values differ significantly from random for all four networks. In general, clusters with high HINscore or pathway homogeneity are homogeneous in terms of associated diseases, meaning that the genes are annotated to similar diseases. For example, gene products of cluster B.1 depicted in Fig. 3, which corresponds to mitochondrial respiratory chain deficiencies and Alexander and Leigh Disease, physically interact and hence the HINscore is very high. In addition, they are all annotated to the same pathway resulting in a pathway homogeneity value of 1. In contrast, clusters with very low homogeneity values (<0.25) are heterogeneous in terms of disease annotation. These clusters, which are underrepresented in the dataset, contain genes with very high allelic heterogeneity. In CURATED, for instance, genes having more than 20 associated diseases, belong to heterogeneous clusters with low pathway homogeneity values (mean = 0.28, sd = 0.11). Thus, the genes in these clusters are annotated to different biological pathways. It could be argued that such genes encode multifunctional proteins that participate in different biological processes, and mutations in these proteins affecting different functions can then lead to different disease phenotypes. This set of genes might be classified as pleiotropic genes [39] or represent genes that “moonlight” between different functions [40]. The diversity of functional annotation of these genes might be “obscuring” the modularity of the associated diseases. Thus, it would be interesting to further investigate the role of these proteins with respect to disease development. Nevertheless, the majority of clusters show medium range HINscore and pathway homogeneity values, suggesting that not a single biological process but a core-set of biological processes is relevant for the disease. This has important implications for disease treatment and drug development. If a disease is associated with several pathways, a therapy considering the diversity of biological processes could be of advantage [41]. And if a set of diseases is related to the same pathways, a treatment already successful for one of the diseases could also be applied to the other diseases [42].

We studied the concept of modularity from the disease and the gene centric perspectives. All in all, our results show that for most diseases, and even for clusters of related diseases, functional modules do exist. Moreover, we show that phenotypically related gene clusters resemble functional modules. Hence, these functional modules can be studied more deeply to shed light on the mechanisms related to the diseases. We therefore determined the specific biological processes relevant for each gene cluster by calculating GO term and pathway enrichment (see Text S1, Section 2.3). We obtained significant (p-value<0.05) GO and pathway enrichment for 94% of the clusters in CURATED. Details on the clusters and the enrichment results are also available online.

There are some limitations to our analysis related to the incompleteness of our databases due to natural limitations in the curation process of the original databases, and related to inaccuracies derived from text-mining. Moreover, annotation issues have to be considered, such as the incomplete annotation of genes to GO terms, biological pathways and HIN, and the incomplete coverage of cross-talks and other annotation issues in pathway databases [43]. Even taking into account the aforementioned limitations, to the best of our knowledge, this is the first global analysis of human genetic diseases including mendelian, complex and environmental diseases. Overall, we observe good quality of text-mining derived associations, as values for LHGDN are comparable to the networks derived from expert-curated databases.

### Case studies

Our comprehensive database represents a suitable framework to study human diseases with genetic origin and also the influence of environmental factors, such as drugs. In this section, we provide some examples making use of our integrated database and the results of the enrichment analysis to inspire future studies and to encourage other researchers to use DisGeNET. Illustrative case studies for the (i) analysis of mechanisms underlying adverse drug reactions, (ii) prediction of disease candidate genes, (iii) study of the interactions between environmental factors and diseases at the genetic level, and (iv) identification of shared mechanisms of distinct diseases are presented. Moreover, we provide a Cytoscape session file including all examples discussed in this article (http://ibi.imim.es/DisGeNET/data/DisGeNET.cys).

#### Case study 1: Analysis of mechanisms underlying adverse drug reactions

**Figure 4:**
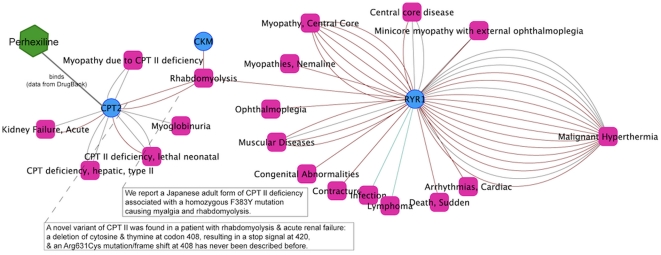
Knowledge about genetic basis of diseases can shed light on mechanisms underlying drug adverse reactions. A network of genes and diseases around Rhabdomyolisis is displayed. The drug Perhexiline is used for treatment of Angina Pectoris and has as therapeutic target CPT13. In addition, it can also target CPT2. Since deficiencies in CPT2 function are associated with Rhabdomyolisis, it can be proposed that Perhexiline causes Rhabdomyolisis through its action on CPT2.

Rhabdomyolysis can result from a traumatic injury, but also appears as a consequence of other diseases or due to intoxication with recreational and prescription drugs, such as Perhexiline. One of the three genes associated with Rhabdomyolysis is CPT2, which encodes the mitochondrial carnitine palmitoyltransferase II (see Fig. 4). Inherited deficiencies in this enzyme lead to CPT2 deficiency, an autosomal recessive disorder characterized by recurrent Myoglobinuria, episodes of muscle pain, stiffness, and Rhabdomyolysis. On the basis of this knowledge it is possible to create a hypothesis on the mechanisms by which certain drugs such as Perhexiline can lead to Rhabdomyolysis. Perhexiline, which is prescribed for severe Angina Pectoris [44] inhibits CPT1, shifting myocardial substrate utilization from long chain fatty acids to carbohydrates (http://www.drugbank.ca/drugs/DB01074). Perhexiline can also target, to a lesser extent, CPT2 [45], which would explain the toxic effects of the drug in skeletal muscles due to the association of CPT2 with Rhabdomyolysis. Note that the association of CPT2 with Rhabdomyolysis was not available in any of the curated databases but in our integrated data set. This example shows the potential of using our gene-disease data in combination with drug-target data for the analysis of drug adverse reactions.

#### Case study 2: Gene clusters and pathway analysis to predict new disease candidate genes

**Figure 5:**
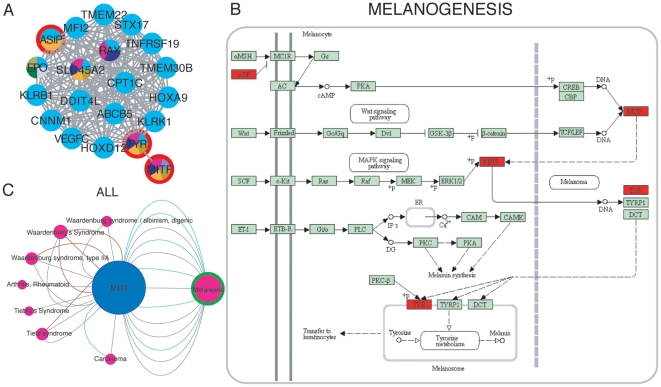
Candidate disease gene prediction. A: Phenotypically derived gene cluster associated with Melanoma. MITF is the only gene in the cluster not associated with Melanoma. B: The Melanogenesis pathway (KEGG: hsa:04916) with genes MITF, TYR and ASP (ASIP in A) colored in red. C: Neighborhood of MITF gene in network ALL.

One of the gene clusters is composed of 20 genes associated with a variety of diseases such as melanoma and developmental diseases affecting pigmentation, eye and ear functions (Tietz and Waardenburg syndromes). Most genes of the cluster are associated with melanoma with the exception of MITF (see Fig. 5A). GO enrichment analysis resulted in terms like “melanin biosynthetic process from tyrosine” (GO:0006583), “eye pigment biosynthetic process” (GO:0006726) and “melanocyte differentiation” (GO:0030318), among others. These are all processes relevant to skin, hair and eye pigmentation, hearing function in the cochlea, and skin carcinogenesis. Fig. 5B shows the Melanogenesis pathway (KEGG hsa:04916), which is the most significantly enriched pathway for this cluster. The proteins encoded by genes TYR and ASIP (ASP in KEGG) and the transcription factor encoded by MITF regulating expression of the TYR gene, are highlighted in red in the pathway. Since MITF appears not only in the same phenotypically derived cluster but also in the same pathway as the genes associated with Melanoma, it could be proposed that MITF is a candidate disease gene for Melanoma. In fact, we could confirm this finding by checking the disease neighborhood of MITF in the gene-disease network (ALL) that also includes text-mining derived information (see Fig. 5C). The information extracted by text-mining indicates that MITF has been reported as a gene involved in melanocyte development and characterized as melanoma oncogene [29], [30]. In conclusion, clustering analysis of the gene projection network followed by functional enrichment analysis can be used to propose new candidate disease genes. We would also like to mention here that the CURATED set of DisGeNET represents a suitable dataset to benchmark computational methods for the prediction of candidate disease genes.

#### Case study 3: Interaction between environmental exposure with arsenic compounds and cancer at the genetic level

Another gene cluster contains 67 genes mostly associated with Arsenic Poisoning, skin and nervous system diseases, and different types of neoplasm. Arsenic is a well established human carcinogen, and many studies support an association between arsenic exposure and increased incidence of solid tumors, such as lung, bladder, prostate, renal and skin tumors [46], [47], [48], [49], [50], [51]. Moreover, studies conducted in developing countries show a general increase in the incidence of different types of cancers, which is hypothesized to be associated with exposure to environmental toxins, among other factors, some of them of genetic origin [52], [53], [54]. Thus, there is a need to investigate the interactions among environmental carcinogens and genetic factors [53]. Although more studies are needed to determine a linkage between arsenic exposure and Breast Cancer incidence [55], this cluster indicates a possible association at the genetic level. Some of the genes that are associated with Arsenic Poisoning are also known to be associated with Breast Cancer, such as TNF, CCL20, CXCL2, CXCL3 and IL1B. Apoptosis-inducing factors IL1B and TNF are down regulated by arsenic compounds [56], as indicated by the supporting evidence of one of the associations in the dataset. This observation combined with the knowledge on DNA damaging effect of arsenic [57] may provide a mechanistic hypothesis for the tumorigenic effects of arsenic.

All in all, cluster analysis of the gene projection network uncovered an interesting relationship between environmental exposure to arsenic compounds and breast cancer. This relationship deserves further investigation at the epidemiological and molecular levels.

#### Case study 4: Identification of shared mechanisms of different diseases

**Figure 6:**
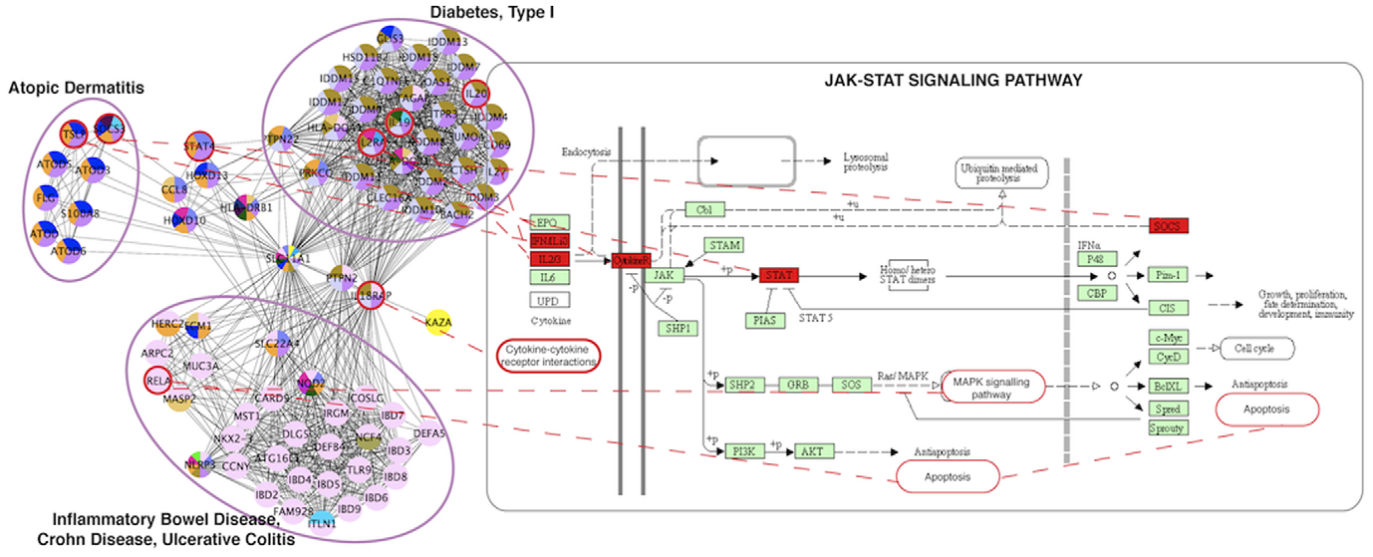
Identification of shared mechanisms of different diseases. A cluster containing genes associated with distinct diseases is shown on the left part of the figure. There are three main disease groups, Atopic Dermatitis (an autoimmune skin disease), Diabetes Mellitus Type I (an early onset, insulin-dependent, autoimmune disease), and Inflammatory Bowel Diseases (including Crohn Disease and Ulcerative Colitis). Diseases are coloured according to their disease class (see Fig. S4). The most significantly enriched Jak-STAT signaling pathway is displayed with some nodes from the cluster colored in red (right part).

Another cluster containing 79 genes is an example of a heterogeneous cluster in which genes are associated with different diseases. Fig. 6 shows the three main disease groups, Atopic Dermatitis (an autoimmune skin disease), Diabetes Mellitus Type I (an early onset, insulin-dependent, autoimmune disease), and Inflammatory Bowel Diseases (including Crohn Disease and Ulcerative Colitis). All these diseases are related as they share many gene associations. Interestingly, according to MeSH, one of the diseases (Crohn Disease) is not classified as Immune Systems Disease but only as Digestive Systems Disease (genes colored in pink). However, it is well established that Crohn Disease is an autoimmune disease [58], [59], and this grouping is captured by the network analysis.

GO term and pathway enrichment analysis showed that for this heterogeneous cluster, there are common biological processes associated with the distinct diseases. For instance, although there are 59 pathways annotated to this cluster, the pathway homogeneity is 41% indicating that almost half of the gene products appear in the same pathway. The most significantly enriched processes are related to immune (GO:0006955) and inflammatory response (GO:0006954), while the most significantly enriched pathway is the Jak-STAT signaling pathway (KEGG hsa:04630). Fig. 6 shows the Jak-STAT signaling pathway, which contains genes associated with all three diseases of this cluster. Interestingly, the connections of the different diseases can be seen on different levels of this signaling pathway, from receptor-ligand interactions towards downstream signaling and transcriptional regulation. This example shows the value of clustering and subsequent GO and pathway enrichment analysis to identify mechanisms that are common to different diseases.

### Conclusions

The first goal of this study was to develop a comprehensive resource covering the current knowledge on human genetic diseases and to provide it to the community. The second goal of this study was to address the concept of modularity in all human diseases with genetic origin. Although in the biomedical literature it is often assumed that human diseases are modular, the validity of this hypothesis has not been tested before in a systematic manner for all diseases with genetic origin. We have performed a detailed study of the emergent properties of human gene-disease networks by means of computational analysis, covering the whole spectrum of human diseases with genetic origin including monogenic, complex and environmental diseases. The results indicate a highly shared genetic origin of monogenic, complex and environmental diseases. Moreover, most diseases cannot be attributed to a single gene but to defects of several genes. Interestingly, these genes are likely to participate in a core set of biological processes. This is even observed in several mendelian disorders, contrasting to previous findings. More strikingly, similar findings are obtained when studying groups of diseases. This suggests that the diseases in these groups, which can be very similar but also very unrelated, might arise due to dysfunction of the same biological processes in the cell. Finally, we identified the core biological processes associated to the diseases in DisGeNET and show in several examples the value of such analysis to unveil the mechanisms leading to disease phenotypes and adverse drug reactions. Our computational analysis has important implications for understanding disease mechanisms and how environmental factors, such as drugs, influence human health. Finally, we provide all gene-disease networks in a user-friendly way through DisGeNET [28], a Cytoscape plugin and as SQLite database for direct computational access to aid future research of disease-related processes that will also benefit drug discovery and development.

## Methods

### Data integration

DisGeNET, a comprehensive database of gene-disease associations was developed by integrating information from four repositories: Online Mendelian Inheritance in Man (OMIM) [6], UniProt/SwissProt (UNIPROT) [60], Pharmacogenomics Knowledge Base (PHARMGKB) [61], and Comparative Toxicogenomics Database (CTD) [8]. In addition, associations from a literature-derived human gene-disease network (LHGDN) [62] were included to increase the coverage of the database. For a correct integration of gene-disease association data, we developed a gene-disease association ontology (see Fig. S1). The data sources, gene-disease association ontology and data integration approach are described below.

### Data sources

OMIM: Online Mendelian Inheritance in Man (OMIM) focuses on inherited diseases. Gene-disease associations were obtained by parsing the mim2gene file for associations of type “phenotype” (data was downloaded from ftp://ftp.ncbi.nlm.nih.gov/gene/DATA/mim2gene on June, 6th 2009) and classified as Marker in our gene-disease association ontology (see Fig. S1). In total, we obtained for 2198 distinct genes and 2473 distinct disease terms 3432 gene-disease associations. After mapping of disease vocabularies, the OMIM network contained 2417 distinct diseases.

UNIPROT: UniProt/SwissProt is a database containing curated information about protein sequence, structure and function. Moreover, it provides information on the functional effect of sequence variants and their association to disease. We extracted this information from UniProt/SwissProt release 57.0 (March 2009) as described in [63]. All protein identifiers were converted to Entrez Gene identifiers in order to allow integration with the other data sources. All gene-disease associations were classified as GeneticVariation. UniProt provided 1746 distinct gene-disease associations for 1240 distinct genes and 1475 distinct diseases.

PHARMGKB: The Pharmacogenomics Knowledge Base (PharmGKB) is specialized on the knowledge about genes that are involved in modulating drug response (pharmacogenes). Genes are classified as pharmacogenes because they are (i) involved in the pharmacokinetics of a drug (how the drug is absorbed, distributed, metabolized and eliminated) or (ii) the pharmacodynamics of a drug (how the drug acts on its target and its mechanisms of action) [61]. Hence, it covers less broadly human gene-disease associations but was found to be complementary to the other sources, as it contains some gene-disease associations not present in the other repositories. We downloaded genes.zip, diseases.zip and relationships. zip from http://www.pharmgkb.org/resources/downloads_and_web_services.jsp on June 6th 2009 and parsed the files to extract gene-disease associations. We furthermore made use of the Perl webservices to obtain all available annotations and supporting information. We included 1772 associations for 79 distinct genes and 261 distinct diseases. PharmGKB associations were classified as Marker if the original label was “Related” and as RegulatoryModification if the original label was “Positively Related” or “Negatively Related”.

CTD: The Comparative Toxicogenomics Database (CTD) contains manually curated information about gene-disease relationships with focus on understanding the effects of environmental chemicals on human health. We downloaded the CTD_gene_disease_relations.tsv file from http://ctd.mdibl.org/downloads/ on June 2nd 2009 and parsed it for gene-disease associations of type “marker” or “therapeutic” (see http://ctd.mdibl.org/help/glossary.jsp for description of the original labels). CTD includes associations from OMIM but with some differences (i) for some associations extra information such as cross-links to PubMed are available and (ii) some associations are missing in either of the two databases. Hence, we kept all available gene-disease associations from both sources. All CTD gene-disease associations were classified as Marker if the original label was “marker” and as Therapeutic if the original label was “therapeutic”. All cross-links to PubMed were kept. In total CTD data provided 6469 associations for 2702 distinct diseases and 3345 distinct genes.

LHGDN: The literature-derived human gene-disease network (LHGDN) is a text mining derived database with focus on extracting and classifying gene-disease associations with respect to several biomolecular conditions. It uses a machine learning based algorithm to extract semantic gene-disease relations from a textual source of interest. The semantic gene-disease relations were extracted with F-measures of 78 (see [62] for further details). More specifically, the textual source utilized here originates from Entrez Gene's GeneRIF (Gene Reference Into Function) database [64]. This database represents a rapidly growing knowledge repository and consists of high-quality phrases created or reviewed by MeSH indexers. Hereby, the phrases refer to a particular gene in the Entrez Gene database and describe its function in a concise phrase. Using this textual repository for text mining has recently gained increasing attention, due to the high quality of the provided textual data in the GeneRIF database [62], [65], [66]. LHGDN was created based on a GeneRIF version from March 31st, 2009, consisting of 414241 phrases. These phrases were further restricted to the organism Homo sapiens, which resulted in a total of 178004 phrases. We extracted all data from LHGDN and classified the original associations using our ontology. In total, LHGDN provided 59342 distinct gene-disease associations for 1850 diseases and 6154 distinct genes. The LHGDN is also available in the Linked Life Data Cloud (http://linkedlifedata.com/sources).

### Gene-disease association ontology

For a correct integration of gene-disease association data, we developed a gene-disease association ontology (see Fig. S1). The GeneDiseaseAssociation ontology describes the different types of association between a gene and a disease and was developed to integrate information from the different databases used in this study. We inspected the different types of gene-disease associations included in each of the databases and developed the ontology as a mean to harmonize them in a common framework. While most of the databases focus on describing “true” associations between genes and diseases, some of the databases also contain information of genes that are known to be not associated to a certain disease phenotype. Thus, an upper level classification of the associations based on the level of certainty of the gene-disease association was used. The “Association” class indicates that there is indeed an association between a gene and a disease; and the “NoAssociation” class indicates that there is no association between a gene and a certain disease state. This can also be expressed as independence between a certain state of the gene/protein and the disease state. Two data sources (PharmGKB and LHGDN) provide associations that were mapped to the class “NoAssociation”, in particular associations labeled “not related” from PharmGKB and “negative association” from LHGDN.

Consequently, we classified all association types as found in the original source databases into *Association* if there is a relationship between the gene/protein and the disease, and into *NoAssociation* if there is no association between a gene/protein and a certain disease (in other words, if there is evidence for the independence between a gene/protein and a disease). In this study, we only considered gene-disease associations of type *Association*. The ontology is available at http://ibi.imim.es/DisGeNET/DisGeNETweb.html#Download.

### Mapping of disease vocabularies and disease classification

We used the Medical Subjects Headings (MeSH) hierarchy for disease classification (see http://www.nlm.nih.gov/mesh/). The repositories of gene-disease associations use two different disease vocabularies, MIM terms (used by OMIM, UniProt, CTD) and MeSH terms (used by CTD, PharmGKB, LHGDN). We used the UMLS metathesaurus to map from MIM to MeSH vocabularies. This step was performed to merge disease terms representing the same disorder, thus reducing redundancy. We were able to map 497 MIM terms directly to MeSH using UMLS and we additionally mapped 23 MIM terms by using a string mapping approach. Briefly, we searched the UMLS metathesaurus for MeSH terms for which there is at least one synonym exactly matching one of the synonyms describing the MIM term of interest. The resulting 63 matched terms were manually checked and reduced to 23 terms. For disease classification, we considered all 23 upper level concepts of the MeSH tree branch C (Diseases), plus two concepts (“Psychological Phenomena and Processes” and “Mental Disorders”) of the F branch (Psychiatry and Psychology). Moreover, we added one disease class “Unclassified” for all disease terms for which a classification was not possible. We categorized all diseases into one or more of the 26 possible disease classes. For MeSH disease terms we directly used its position in the MeSH hierarchy, for MIM disease terms not mapped to MeSH, we used the disease classification of [22]. Then, we mapped their disease classification to the MeSH hierarchy and extended the mapping using a disease classification available at CTD (CTD_disease_hierarchy.tsv downloaded August, 8th 2009). In total, we were able to classify 3980 (98.39%) diseases. The disease classification allows filtering and searching of the network restricted to disease class, all implemented within DisGeNET [28]. The disease classification is available as text file in the supplementary material (Text S2). Many diseases are assigned to more than one disease class as several systems or organs are affected. Fig. S4 shows the disease color mapping used in DisGeNET [28]. Disease and gene nodes can be colored according to their disease class and can have multiple colors if they are assigned to more than one disease class.

### Generation of gene-disease networks

The gene-disease associations can be represented as bipartite graphs, which have two types of vertices and the edges run only between vertices of unlike types [67]. Accordingly, we constructed four different networks, OMIM, CURATED (containing associations from expert curated databases), LHGDN (text-mining derived associations) and ALL (containing all associations). In our networks, multiple edges represent the multiple data sources reporting the gene-disease association. We generated two projections, one for the diseases and one for the genes using the igraph library in R [68]. The projected networks contain only vertices of the same kind (monopartite) and two nodes are connected if they share a neighbor in the original bipartite graph. Before calculating node degree distributions and projecting the networks, we simplified the graphs by removing multiple edges. In the simplified graphs the node degree represents the number of first neighbors.

All gene-disease networks are available as SQLite database and through DisGeNET, a Cytoscape plugin, both available online at http://ibi.imim.es/DisGeNET/DisGeNETweb.html#Download.

### Graph clustering

To identify clusters in the disease and gene projection networks, we used the MCL graph cluster algorithm [69], which has successfully been applied to protein family detection [70]. We applied the algorithm using edge weights calculated as follows:
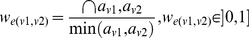
(1)where *e(v1, v2)* is the edge connecting vertices *v1* and *v2* and *a_v_* is the number of annotations to vertex *v* (genes to disease nodes or diseases to gene nodes). Edge weights range from zero to one (excluding zero), where one means that the two vertices share all annotations of the node with less annotation.

### Pathway homogeneity

It has been shown that for OMIM diseases the associated genes are involved in the same biological and cellular processes [17], [22], [71]. In order to test if this concept applies for our integrated data set, we calculated pathway homogeneity for (i) each disease separately, (ii) for the disease clusters and (iii) the gene clusters.

Homogeneity is defined as the maximum fraction of genes sharing the same biological annotation:
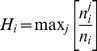
(2)where *n_i_* is the total number of genes in the disease, disease cluster or gene cluster (i) with annotations, and *n^j^_i_* is the number of genes sharing the same biological annotation (j). The pathway annotation was downloaded from KEGG (ftp://ftp.genome.jp/pub/kegg/genes/organisms/hsa) and Reactome (http://www.reactome.org/download/index.html) on November, 11^th^ 2009.

To calculate random controls for pathway homogeneity for single diseases and disease clusters, we randomly sampled genes from the set of disease genes of the studied network with annotation to pathways. We then took the annotation of the corresponding gene products and calculated pathway homogeneity values. Random controls for gene clusters were obtained by randomly assigning genes to clusters while total number of clusters and original cluster sizes were maintained. Random sampling was repeated 10^4^ times to reach statistical significance and averages were compared to real values. We use a significance level of 0.05. Moreover, binning of cluster sizes was performed to show dependence of cluster sizes and homogeneity values, for the bin-wise comparison of mean values, 95% confidence intervals were calculated. To study the correlation between homogeneity values and the number of associated gene products we calculated the Pearson correlation coefficient (r).

### HINscore calculation

To evaluate if disease genes products belonging to our clusters are more likely to interact directly than randomly selected genes we calculated the HINscore using the recently published human interaction network (HIN) [36]. HIN is based on protein-protein interaction data from HPRD and pathway data from Reactome, NCI/Pathway Interaction database and the MSKCC Cancer Cell map. The HINscore for each gene cluster is defined as:
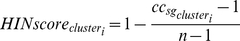
(3)where *cc* is the number of connected components of subgraph *sg* built using all nodes in *cluster_i_* connected by edges appearing in the human interaction network (HIN), and n is the total number of gene products in *cluster_i_*. The HINscore reflects the degree to which the gene products in a cluster are connected between them in terms of direct (physical) interactions. The HINscore is 1 if all nodes in a cluster are directly connected in HIN; it is 0 if they are not connected in HIN but only in the gene cluster due to shared disease associations. Therefore, the HINscore represents a means to measure to what extent the gene clusters represent functional modules and hence to measure the modularity of human diseases. We compared the HINscores with random controls for all four networks. For the random controls, we randomly selected the same number of genes per cluster from the samples set consisting of all genes in the network of study being present in HIN. We repeated the randomization process 10^4^ times to achieve statistical significance. We display mean HINscore for different cluster sizes and 95% confidence intervals. We use a significance level of 0.05.

## Supporting Information

Figure S1 **Gene-disease association ontology.** Gene-disease association ontology developed to allow correct integration of information from diverse repositories.
(TIF)

Figure S2 **Number of distinct gene/disease nodes and edges per data source.** The number of diseases refers to the actual number of disease nodes in the networks after mapping of disease vocabularies. The number of edges (simplified) refers to the number of distinct gene-disease associations. The number of edges (multiple) represents all edges, considering one edge for each source or evidence reporting the gene-disease association.
(TIF)

Figure S3 **Venn diagrams of data overlap among databases.** The upper panel shows the overlaps among the individual expert curated databases. The lower panel displays the overlap of CURATED and the text-mining derived network (LHGDN).
(TIF)

Figure S4 **Disease classes visualization in DisGeNET.** Diseases were classified into 26 disease classes according to the MeSH hierarchy allowing the analysis of groups of related diseases based on standard disease classification. Using this disease classification, many diseases are assigned to more than one disease class as different systems or organs are affected.
(TIF)

Figure S5 **Degree distributions of the bipartite networks.** The node degree distributions of the bipartite networks are plotted showing (A) the number of associated genes per disease and (B) the number of associated diseases per gene. Red arrows highlight the two disease- or gene-nodes with highest degree. Moreover, average degree values are plotted.
(TIF)

Figure S6 **Pathway homogeneity for disease clusters.** Mean pathway homogeneity values for different number of associated gene products are plotted and compared to random controls (CI 95%).
(TIF)

Figure S7 **Pathway homogeneity for gene clusters.** Mean pathway homogeneity values for different number of associated gene products are plotted and compared to random controls (CI 95%).
(TIF)

Text S1 Supplementary material describing topological an functional network analysis and statistics on gene annotations.
(DOC)

Text S2 Disease terms from DisGeNET and their classification according to MeSH (tab separated file).
(TXT)
