# Supplementary figures and images for: Gene-Disease Network Analysis Reveals Functional Modules in Mendelian, Complex and Environmental Diseases
Source: PLoS One. 2011 Jun 14;6(6):e20284. doi: 10.1371/journal.pone.0020284 (PMC3114846; doi:10.1371/journal.pone.0020284)

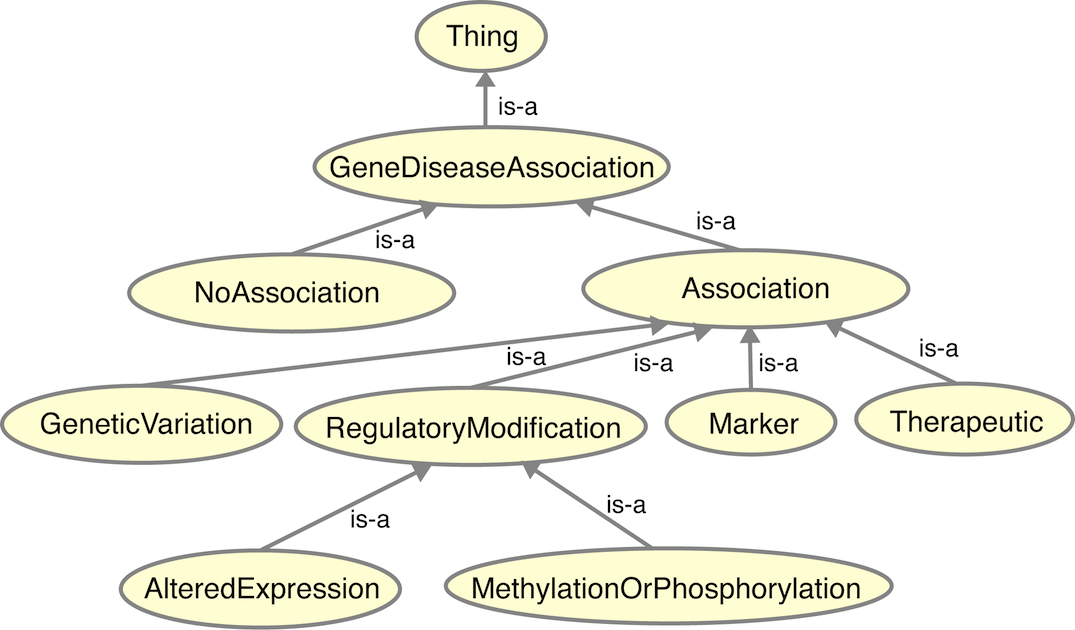

Supplement: Figure S1 — Gene-disease association ontology. Gene-disease association ontology developed to allow correct integration of information from diverse repositories. (TIF) [file pone.0020284.s001.tif]

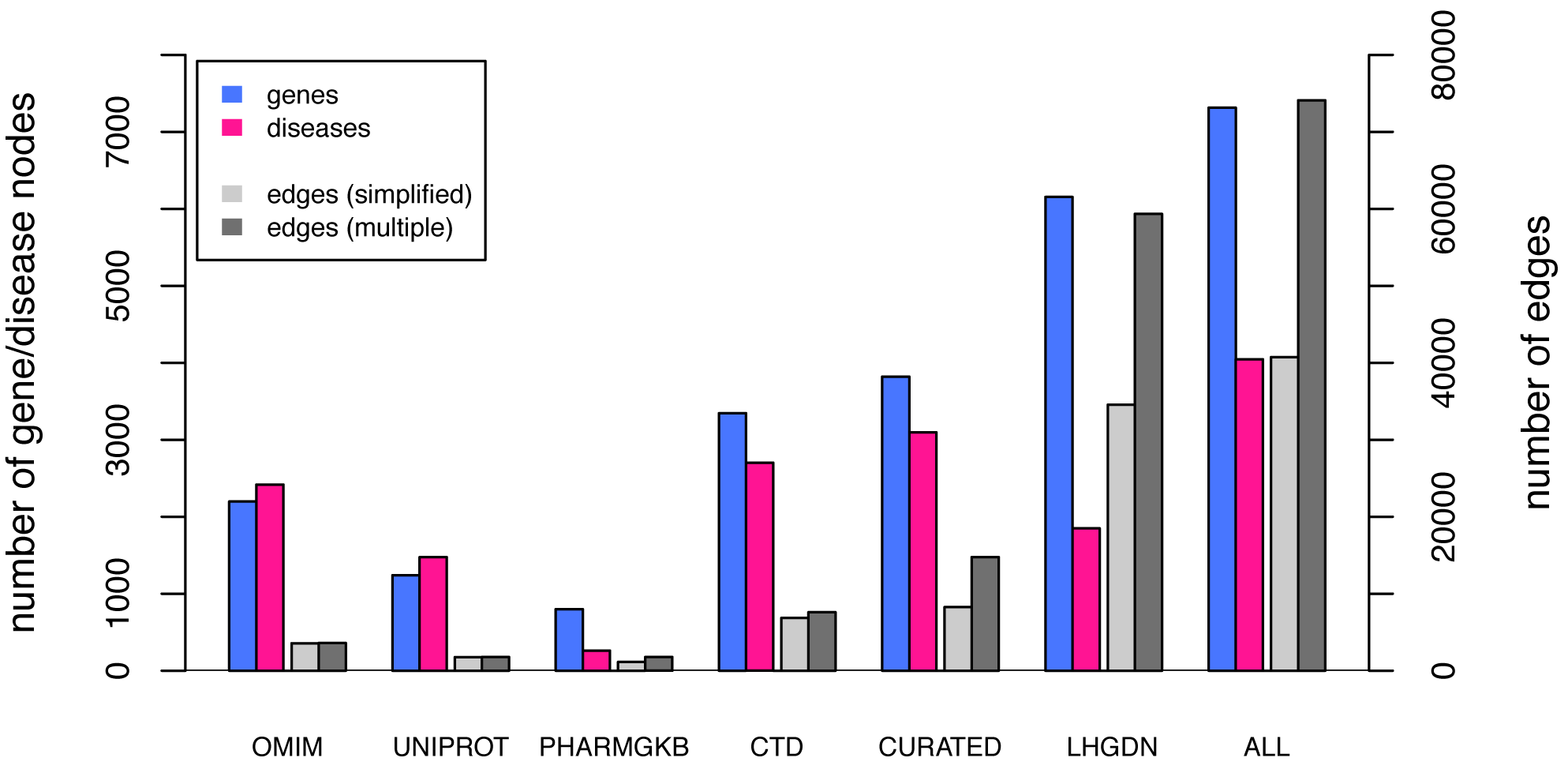

Supplement: Figure S2 — Number of distinct gene/disease nodes and edges per data source. The number of diseases refers to the actual number of disease nodes in the networks after mapping of disease vocabularies. The number of edges (simplified) refers to the number of distinct gene-disease associations. The number of edges (multiple) represents all edges, considering one edge for each source or evidence reporting the gene-disease association. (TIF) [file pone.0020284.s002.tif]

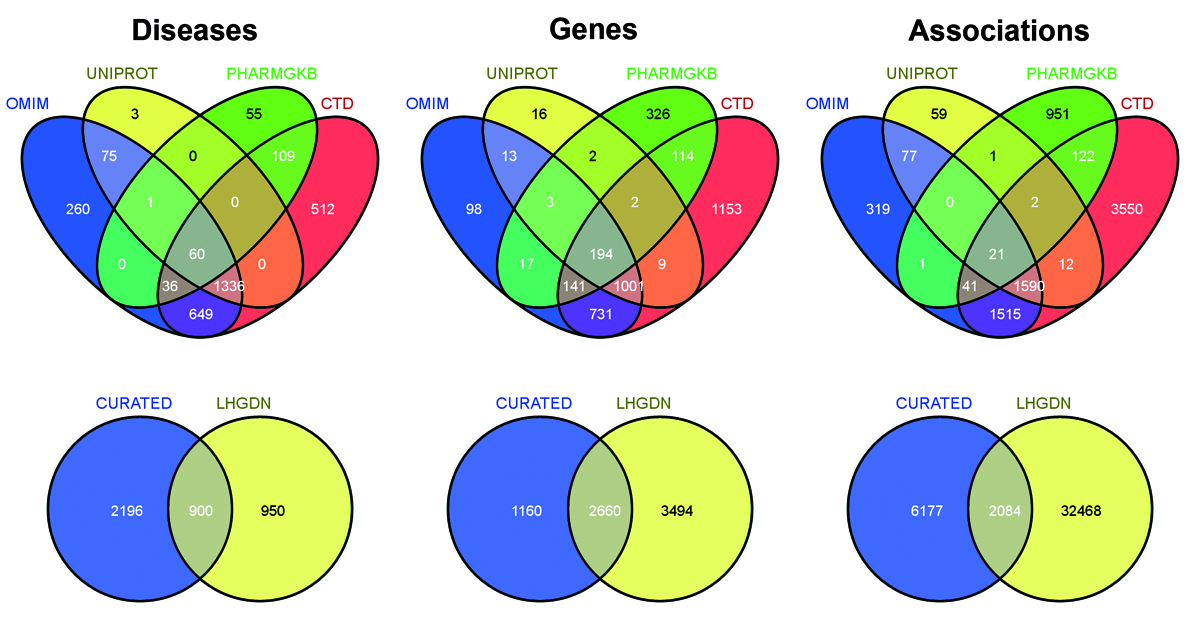

Supplement: Figure S3 — Venn diagrams of data overlap among databases. The upper panel shows the overlaps among the individual expert curated databases. The lower panel displays the overlap of CURATED and the text-mining derived network (LHGDN). (TIF) [file pone.0020284.s003.tif]

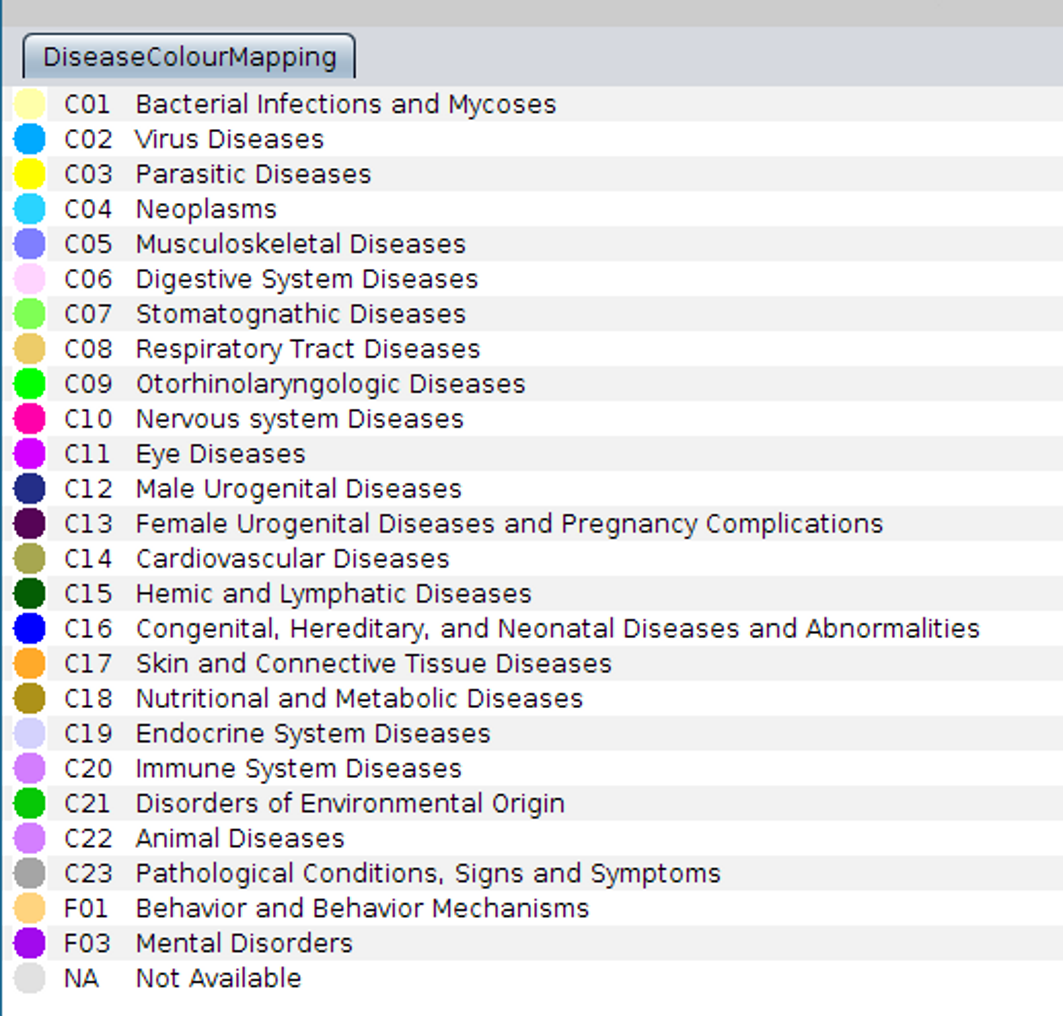

Supplement: Figure S4 — Disease classes visualization in DisGeNET. Diseases were classified into 26 disease classes according to the MeSH hierarchy allowing the analysis of groups of related diseases based on standard disease classification. Using this disease classification, many diseases are assigned to more than one disease class as different systems or organs are affected. (TIF) [file pone.0020284.s004.tif]

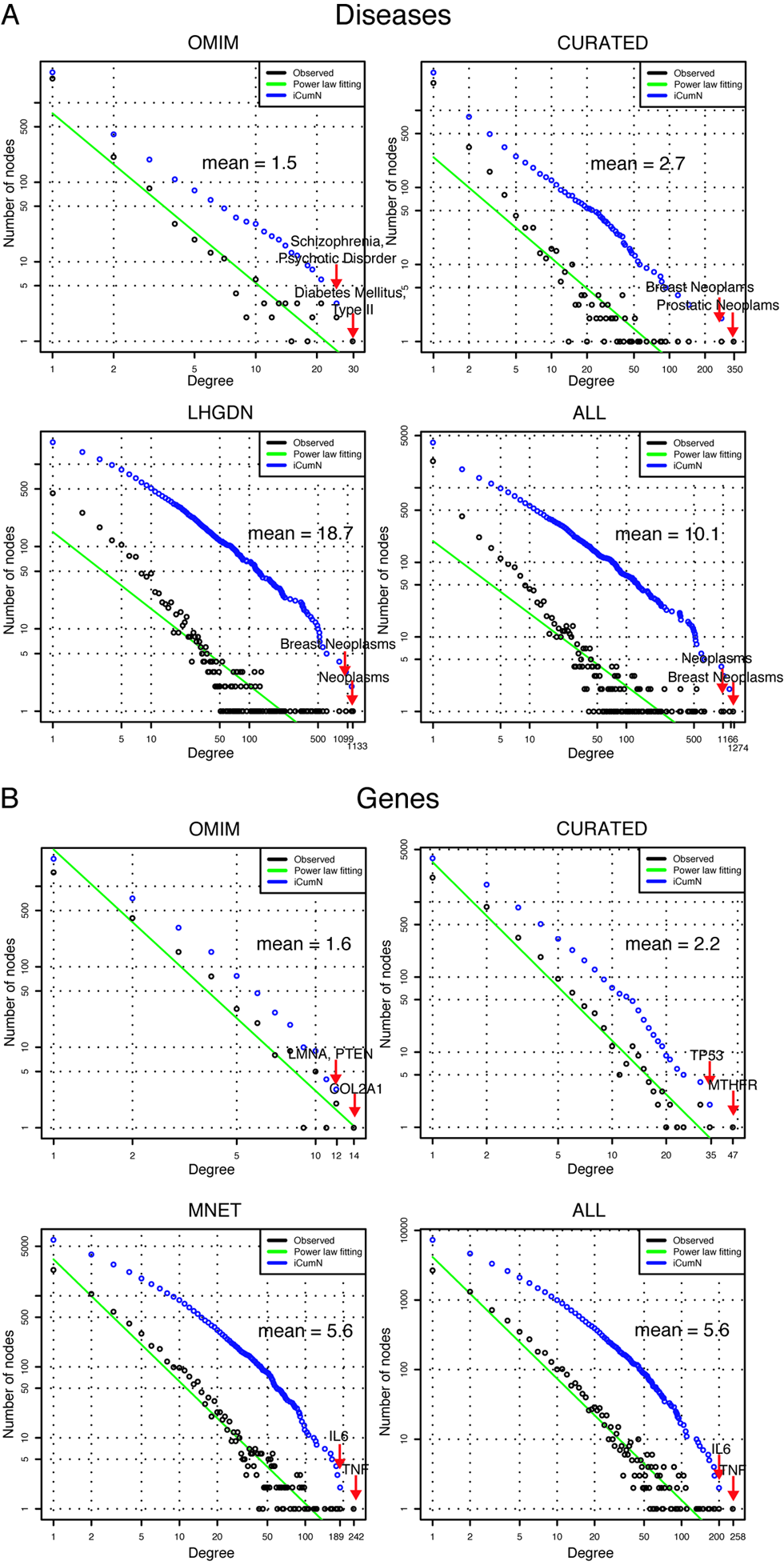

Supplement: Figure S5 — Degree distributions of the bipartite networks. The node degree distributions of the bipartite networks are plotted showing (A) the number of associated genes per disease and (B) the number of associated diseases per gene. Red arrows highlight the two disease- or gene-nodes with highest degree. Moreover, average degree values are plotted. (TIF) [file pone.0020284.s005.tif]

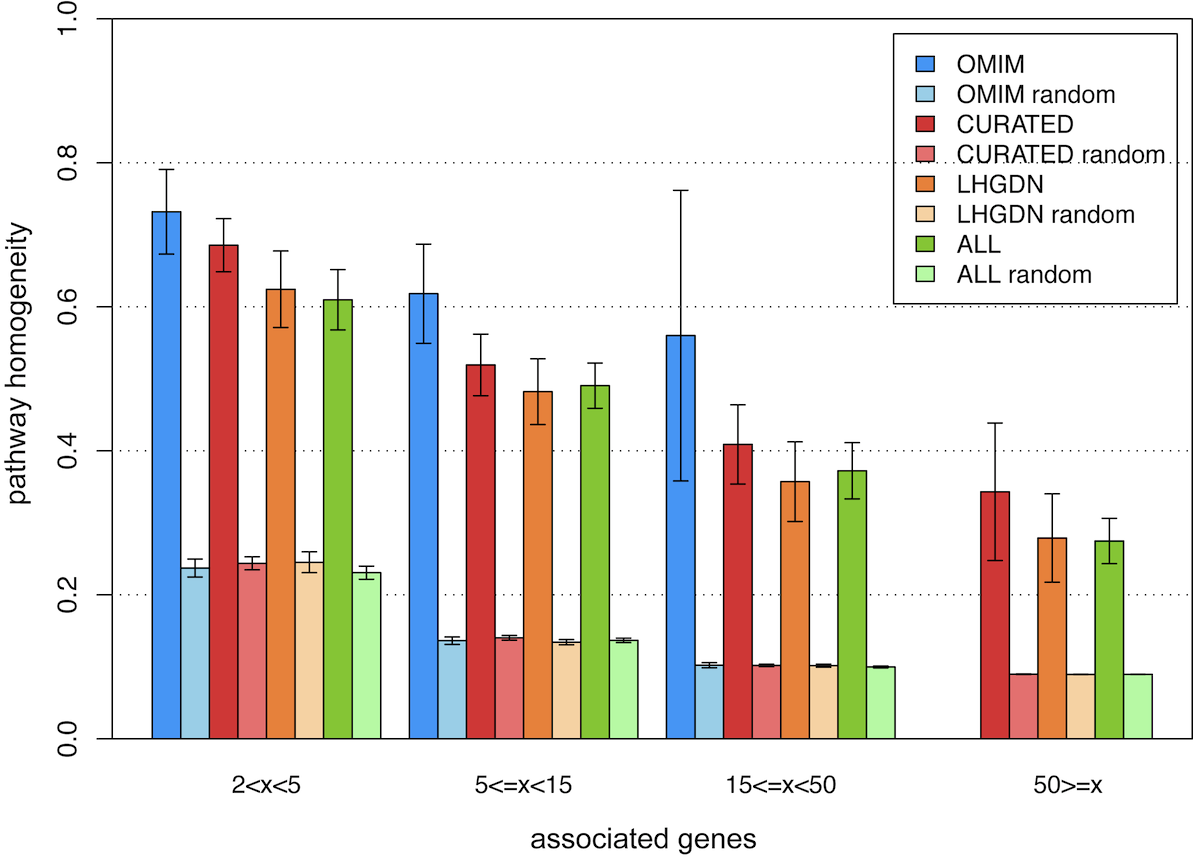

Supplement: Figure S6 — Pathway homogeneity for disease clusters. Mean pathway homogeneity values for different number of associated gene products are plotted and compared to random controls (CI 95%). (TIF) [file pone.0020284.s006.tif]

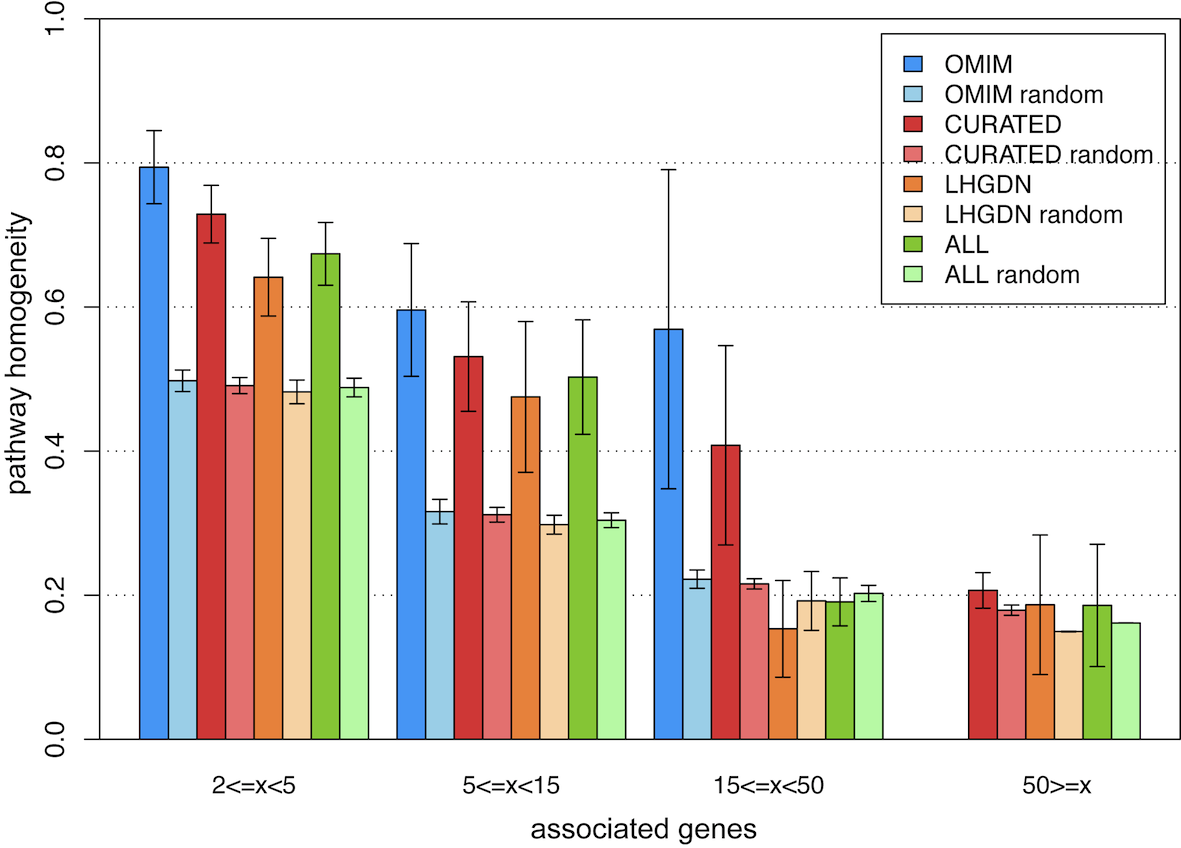

Supplement: Figure S7 — Pathway homogeneity for gene clusters. Mean pathway homogeneity values for different number of associated gene products are plotted and compared to random controls (CI 95%). (TIF) [file pone.0020284.s007.tif]
