# Supplementary material for: Gene-Disease Network Analysis Reveals Functional Modules in Mendelian, Complex and Environmental Diseases
Source: PLoS One. 2011 Jun 14;6(6):e20284. doi: 10.1371/journal.pone.0020284 (PMC3114846; doi:10.1371/journal.pone.0020284)
Supplement: Text S1 — Supplementary material describing topological an functional network analysis and statistics on gene annotations. (DOC) [file pone.0020284.s008.doc]

**Supplementary material for the article**

Gene-disease network analysis reveals functional modules in mendelian, complex and environmental diseases

Anna Bauer-Mehren^1^, Markus Bundschus^2^, Michael Rautschka^1^, Miguel A. Mayer^1^, Ferran Sanz^1^ , Laura I. Furlong^1,3^

^1^Research Programme on Biomedical Informatics (GRIB), IMIM (Hospital del Mar Research Institute), Universitat Pompeu Fabra, Barcelona, Spain

## ^2^Institute for Computer Science, Ludwig-Maximilians-University Munich, Munich, Germany. Current address: Roche Diagnostics GmbH, Penzberg, Germany

## ^3^Corresponding author, Contact: lfurlong@imim.es

[1 Network properties - node degree distributions 2](#__RefHeading___Toc292186302)

[2 Functional analysis 2](#__RefHeading___Toc292186303)

[2.1 Pathway homogeneity – disease clusters 2](#__RefHeading___Toc292186304)

[2.2 Pathway homogeneity – gene clusters 3](#__RefHeading___Toc292186305)

[2.3 GO-BP and pathway enrichment 3](#__RefHeading___Toc292186306)

[3 Gene annotations 3](#__RefHeading___Toc292186307)

[4 References 3](#__RefHeading___Toc292186308)

# Network properties - node degree distributions

Studying the degree distribution of networks allows us to distinguish between different types of networks. For example, random networks show a typical peak corresponding to the average degree in the degree distribution.

In a bipartite graph there exist two degree distributions, one for each vertex type (disease and genes) [1]. The first important observation is that the degree distributions for diseases and genes are different from the degree distribution of random networks, but none of them follow a power law distribution (Fig. S5).

For the diseases, the average node degree increases from 1.5 in OMIM to 10.1 in ALL. The node degree can be used as a measure of the locus heterogeneity of a given disease. There is a dramatic increase in the maximum locus heterogeneity observed in each data set, from 30 genes annotated to Diabetes Mellitus Type II in OMIM, 350 genes associated with Prostatic Neoplasms in CURATED, 1133 genes associated with Neoplasms in LHGDN and 1274 genes associated with Breast Neoplasms in ALL (see Fig. S5). Interestingly, when considering the 10 top-ranking diseases in terms of locus heterogeneity, three diseases in OMIM belong to the “Neoplasm” disease class, 7 in CURATED and 10 in ALL. This may be due to the fact that cancer is one of the most studied diseases and hence more knowledge is available on the relationship of genes and different cancer types.

With respect to the genes, the increase in the node degree is less dramatic but still visible (from an average degree of 1.6 in OMIM to 5.6 in ALL). The degree of the gene in the bipartite graph can be used as a measure of the allelic heterogeneity (the number of diseases associated with a gene). In OMIM, collagen type II alpha 1 (COL2A1) has most disease annotations and there is another collagen, collagen type I alpha 1 in the list of the 10 top-ranked genes. In CURATED, collagen type II is in the top-ranked 30 genes but not for LHGDN or ALL. Moreover, the 10 top-ranked genes of OMIM and CURATED include some cancer related genes such as PTEN and TP53, which is also one of the genes with most disease associations in LHGDN and ALL. The list of the 10 top-ranked genes in CURATED includes cancer related genes (TNF, KRAS) but also many genes related to inflammation such as PTGS2 and IL6. In LHGDN and ALL the 10 top-ranked lists are very similar and contain mainly cancer related genes: TNF, TP53, TGFB1 and genes involved in immune system responses (IL6, IL10, IL1B).

We can also consider the degree distributions of the disease and gene projection networks. In contrast to the bipartite graph degree distribution, the degree of a gene (disease) node indicates the number of gene (disease) neighbors in the gene (disease) projection network. Interestingly, the degree distributions of the projected networks are much broader than the degree distributions of the bipartite graph (data not shown). The right tail of the distributions get much more populated the more data sources are included (more hubs in CURATED than in OMIM and again many more hubs in the LHGDN than in CURATED). Moreover, in the disease projection the average number of diseases connected to any disease is 2.2 in OMIM, 8.5 in CURATED and 103.6 in ALL, suggesting a higher degree of relatedness of human diseases than expected by solely considering a single data source (e.g. OMIM).

In summary, the degree distributions for diseases and genes are different from degree distribution of typical random networks, but none of them follows a power law. Moreover, there is a large dispersion of the right tail that is more evident the more data is incorporated into the networks. There is an increase in the average degree of the nodes, in the number of hubs and also in the degree of the hubs as a consequence of including more information in the network.

# Functional analysis

It has been shown that, for OMIM diseases, the associated genes are involved in the same biological and cellular processes [2,3,4]. In order to test if this concept still applies for our data set, we calculated pathway homogeneity for each disease individually, disease and gene clusters as described in the methods section.

## Pathway homogeneity – disease clusters

We calculated pathway homogeneity for our disease clusters derived from graph clustering of the disease projection networks. Overall, we obtained similar results as for individual diseases. Fig. S6 shows the average pathway homogeneity values of disease clusters plotted for different sizes of associated gene products. Similarly to individual diseases, pathway homogeneity decreases with increasing size of associated gene products. On average, pathway homogeneity for OMIM is 0.73 (sd 0.24) and 0.69 (sd 0.25) for CURATED suggesting that 69 - 73% of the gene products belonging to a disease cluster participate in the same pathway. For the larger networks (LHGDN and ALL) the average pathway homogeneity values of disease clusters slightly decreases to approximately 0.48 (sd 0.23). All values are significantly different from random (p-value < 0.05).

## Pathway homogeneity – gene clusters

We calculated pathway homogeneity for gene clusters. Fig. S7 shows average pathway homogeneity values for different cluster sizes. Here, the cluster size refers to the number of associated gene products of the cluster with annotation to pathways. In CURATED, up to cluster size 50, the average pathway homogeneity is significantly (p-value < 0.05) higher with respect to randomly selected clusters. On average, 77.5% of the clusters have a GO-BP homogeneity value larger than 0.5 or higher; hence more than half of the genes are annotated to the same biological pathway.

## GO-BP and pathway enrichment

For the functional enrichment analysis, we used the R package GOstat [5] and calculated for each gene cluster in CURATED the enrichment of terms in each GO category (biological process, molecular function, cellular component), as well as enriched pathways (KEGG). As reference background we used the list of disease genes that have at least one term annotated. We applied conditional hypergeometric test using a p-value cut-off of 0.05 and restricted the result to terms for which there were at least two genes annotated to in the reference background. The annotation of gene ids to GO terms was taken from the annotation package “org.Hs.eg.db” based on data provided by Entrez Gene (<ftp://ftp.ncbi.nlm.nih.gov/gene/DATA/>) with a date stamp of September 2009. Annotation to pathways was taken from “KEGG.db” with mappings to pathways from KEGG Genome of March 2009. We only calculated GO term and pathway enrichment for clusters containing more than 3 genes.

# Gene annotations

We used annotation of genes to GO-BP, pathways and HIN. Table 1 shows the number of disease genes per network that actually have annotation to GO-BP, pathways or were part of HIN.

Table 1: GO and pathway annotation

| With annotation to | OMIM  (2198) | CURATED  (3820) | LHGDN  (6154) | ALL  (7314) |
| --- | --- | --- | --- | --- |
| GO-BP | 2117 | 3417 | 5704 | 6460 |
| Pathway (KEGG and Reactome) | 1249 | 2007 | 3271 | 3620 |
| HIN | 1628 | 2685 | 4670 | 5175 |

# References

1. Newman MEJ (2003) The structure and function of complex networks. SIAM Review 45: 167-256.

2. Hartwell LH, Hopfield JJ, Leibler S, Murray AW (1999) From molecular to modular cell biology. Nature 402: C47-52.

3. Ravasz E, Somera AL, Mongru DA, Oltvai ZN, Barabási AL (2002) Hierarchical organization of modularity in metabolic networks. Science 297: 1551-1555.

4. Goh K-I, Cusick ME, Valle D, Childs B, Vidal M, et al. (2007) The human disease network. Proc Natl Acad Sci 104: 8685-8690.

5. Falcon S, Gentleman R (2007) Using GOstats to test gene lists for GO term association. Bioinformatics 23: 257-258.
